# Platelet versus plasma CXCL14, coronary artery disease, and clinical outcomes

**DOI:** 10.1016/j.rpth.2023.100165

**Published:** 2023-04-25

**Authors:** Christoph Schories, Peter Martus, Tianyun Guan, Jessica Kristin Henes, Alexander Witte, Karin Müller, Tobias Geisler, Madhumita Chatterjee, Meinrad Gawaz, Dominik Rath

**Affiliations:** 1 Department of Cardiology and Angiology, University Hospital Tübingen, Tübingen, Germany; 2 Institute for Clinical Epidemiology and Applied Biostatistics, University Hospital Tübingen, Tübingen, Germany; 3 Department of Cardiology, the Second Hospital of Jilin University, Jilin, People’s Republic of China; 4 Department of Pharmacology, Experimental Therapy and Toxicology, University Hospital Tübingen, Tübingen, Germany

**Keywords:** coronary artery disease, human CXCL14 protein, left ventricular function, platelets, prognosis

## Abstract

**Background:**

Platelets express CXCL14, while platelet-derived CXCL14 induces monocyte chemotaxis and exerts an angiostatic effect on endothelial cells.

**Objectives:**

This study investigated both platelet surface–associated and circulating levels of CXCL14 in patients with heart disease and associations of this chemokine with myocardial function and outcomes in patients with coronary artery disease (CAD).

**Methods:**

This prospective study enrolled 450 patients with symptomatic heart disease. Platelet surface–associated and plasma CXCL14 levels were analyzed. All patients were followed up for 360 days for a primary composite outcome consisting of all-cause mortality, myocardial infarction, and/or ischemic stroke. Secondary outcomes consisted of the single events of all-cause mortality or myocardial infarction.

**Results:**

Baseline platelet-associated but not circulating CXCL14 levels were significantly lower in patients with chronic coronary syndrome (mean fluorescence intensity logarithmized, 1.35 ± 0.35) when compared to those with acute coronary syndrome (1.47 ± 0.38) and without CAD (1.51 ± 0.40). Platelet CXCL14 levels were significantly lower (1.37 ± 0.37 vs 1.48 ± 0.39) and circulating CXCL14 levels were significantly higher (lg, 2.88 ± 0.20 pg/mL vs 2.82 ± 0.26 pg/mL) in patients with normal baseline left ventricular ejection fraction (LVEF) when compared to those with impaired LVEF. Low baseline circulating CXCL14 (hazard ratio, 2.33; 1.00-5.46) but not platelet CXCL14 was associated with worse outcome in patients with CAD.

**Conclusion:**

Platelet-associated and circulating CXCL14 levels show differential regulation in patients with and without CAD. Although platelet-associated CXCL14 increased and circulating CXCL14 decreased with impairment of LVEF, only lower circulating CXCL14 upon admission was associated with worse prognosis in patients with CAD.

## Essentials

• CXCL14 may be associated with recovery of myocardial function in coronary artery disease (CAD).
• We investigated both platelet and circulating CXCL14 in 450 patients with heart disease.
• CXCL14 differed significantly between patients with CAD and those without CAD and correlated with myocardial function.
• Low circulating CXCL14 levels were associated with worse outcomes in patients with symptomatic heart disease.

## 1 Introduction

Besides their role in thrombosis and hemostasis, platelets are critically involved in inflammatory and immunomodulatory processes and thus progression of atherosclerosis [1]. Platelets store a variety of chemokines, which are released upon activation. Acting as both autocrine and paracrine mediators, they potentially promote inflammation in their immediate microenvironment and, thus, atherosclerosis or participate in its resolution [2]. Some platelet-derived chemokines like CXCL14 [[3], [4], [5]] and PF4 [6] are angiostatic, while SDF-1α [6] is both angiogenic and regenerative [7]. Therefore, activated platelet–derived mediators may decide on the delicate balance between vascular inflammation and regeneration [3,4,8]. The chemokine CXCL14 (BRAK, BMAC, Mip-2γ) has been shown to be expressed in leukocytes, endothelial cells [3], as well as in both human and murine platelets at protein levels and secreted upon activation [4]. CXCL14 expression in cells can be governed by genetic modulation in terms of methylation state and single-nucleotide polymorphisms (SNPs), which may have a bearing on its pathophysiologic actions, as recently shown by us in case of junctional adhesion molecule-A (F11R) in patients with coronary artery disease (CAD) [9]. Although CXCL14 SNPs have not been explored in the context of CAD, there are reports from studies on patients with influenza A (H1N1). Influenza infection is associated with increased cardiovascular risk and mortality particularly during the influenza season, also shown to be a predisposing factor for atherosclerosis. Influenza can also be a trigger for acute coronary syndrome (ACS) [10,11]. On the contrary, administration of influenza vaccine can reduce cardiovascular events in patients with CAD [12]. The influence of methylation in the CXCL14 promoter region and SNPs rs2237061, rs2237062, and rs2547 have been assessed with respect to disease severity in patients with influenza A (H1N1). This study revealed that CXCL14 expression is decreased and CXCL14 methylation is enhanced, even more so with respect to disease severity in patients with HIN1 infection, and correlates with decreased number of T lymphocytes. SNP analysis further revealed that although the frequency distribution of genotypes and alleles of rs2237061 and rs2237062 show no significant differences, there is a significant difference observed for rs2547. Compared with the GG–wild-type carriers of rs2547, GA-mutant and AA-mutant carriers show increased risk of H1N1, therefore assigned as risk genotypes for H1N1 [13]. No doubt, similar studies in the context of CAD might reveal important insights.

CXCL14 mRNA is significantly upregulated in the left anterior descending arteries of obese pigs [14]. Furthermore, CXCL14 shows a strong chemotactic effect on monocytes, suggesting a potential role of this chemokine in monocyte migration and differentiation [4,15]. Besides, monocytes represent key players in atherosclerosis since they promote vascular inflammation [16]. This chemotactic influence has also been specifically shown for activated platelet–derived CXCL14 [4]. Platelet-derived CXCL14 imposes inhibition on the proliferation and migratory potential of human umbilical vein endothelial cells in scratch assays *in vitro* [4,17]. Platelet-derived CXCL14, on one hand, may prevent angiogenesis and, on the other hand, may foster atheroprogression in patients with pre-existing atherosclerotic disease.

CXCL14 may also modulate platelet functionality. Thrombus stability is affected in CXCL14-deficient mice, which can be recovered to normal levels upon *ex vivo* supplementation with recombinant CXCL14 [5]. CXCL14-mediated impact on platelet function could be exerted through CXCR4, which probably represents a major receptor for CXCL14, although conflicting evidence both in favor of and against this possibility has been reported [4,5]. Nevertheless, platelets do express CXCR4 [18,19]. We have previously shown that CXCR4 platelet surface exposure is higher in patients with CAD when compared to healthy individuals [18]. Additionally, we have demonstrated that lower platelet CXCR4 is associated with worse prognosis in patients with CAD [20]. We have also shown that recombinant CXCL14–mediated chemotaxis is affected in platelets derived from CXCR4-deficient murine and human induced pluripotent stem cell (iPS) culture–derived CXCR4-negative platelets [5]. This suggests that CXCR4 may well be a functional receptor for CXCL14 in platelets. Considering the potential implications of platelet-derived or platelet-associated CXCL14 and its receptor CXCR4 on functional recovery following myocardial infarction (MI), this study aimed to investigate both platelet surface–associated and circulating levels of CXCL14 in patients with heart disease. We sought to investigate differential expression patterns between CXCL14 levels in patients diagnosed with ACS, chronic coronary syndrome (CCS), and without CAD. Finally, we aimed to assess potential associations of CXCL14 with myocardial function and prognosis in patients with CAD.

## 2 Methods

### 2.1 Patient cohort

This was a prospective study of patients with symptomatic heart disease. In this study, 450 patients were enrolled. The reasons for admission consisted of elective cardiac catheterization and acute chest pain. All patients received cardiac catheterization immediately before study enrollment. Based on the results of cardiac catheterization, the patients were grouped into those having CAD vs those not having CAD. Furthermore, 177 (39.3%) patients were diagnosed with CCS, 211 (46.9%) with ACS, and 62 (13.8%) without CAD. Patients without CAD had valve stenoses and insufficiencies, myocarditis, nonischemic cardiomyopathies, and hypertensive crises. CCS was defined as the “different evolutionary phases of CAD, excluding situations in which an acute coronary artery thrombosis dominates the clinical presentation.” [21] ACS was defined as unstable angina, non–ST-segment elevation MI, or ST-segment elevation myocardial infarction (STEMI) according to current guidelines [22]. All patients were admitted to the Department of Cardiology and Angiology at the University Hospital of Tübingen, Germany, for symptomatic heart disease. Most patients gave written informed consent, and for those in whom this could not be obtained due to logistic issues, the institutional ethics committee approved use of data and leftover blood samples from clinical care (478/2022BO2). The study was approved by the ethics committee of the University of Tübingen before enrollment of the first patient (270/2011BO1 and 237/2018BO2, respectively). The study complies with the Declaration of Helsinki and Good Clinical Practice guidelines.

### 2.2 Blood collection

Ten milliliters of blood was collected at the cardiac catheterization laboratory from the arterial sheath in the femoral or radial artery during cardiac catheterization.

### 2.3 Flow cytometry

Platelets in whole blood were analyzed for surface expression of CD62P, CXCR4, and CXCL14, gating for the platelet-specific marker CD42b. Blood was collected in citrate phosphate dextrose adenine, diluted 1:50 with phosphate-buffered saline (Gibco), and incubated with the fluorochrome-conjugated antibodies anti-CD62P-FITC (Beckman Coulter), anti-human CXCR4-PE (R&D Systems), anti-human CXCL14-FITC (Biorbyt), anti-human CD42b-PE (BD Biosciences), and anti-human CD42b-FITC (Beckman Coulter) for 30 minutes at room temperature. After fixing the cells with 0.5% paraformaldehyde, samples were analyzed via flow cytometry (FACS-Calibur flow cytometer Becton-Dickinson). Flow cytometer settings were adjusted with respect to specified isotype controls for the antibodies used.

### 2.4 Enzyme-linked immunosorbent assay (ELISA)

Plasma CXCL14 levels were detected with a commercially available ELISA kit (Quantikine CXCL14 ELISA from R&D Systems) according to the manufacturer’s guidelines. EDTA blood samples were centrifuged for 15 minutes at 10,000 *g* within 30 minutes of blood collection. Samples were aliquoted and stored at −80 °C until analysis [23].

### 2.5 Impedance platelet aggregometry

We applied the Multiplate analyzer to study platelet aggregation levels. Furthermore, 600 μL of blood samples acquired in hirudinized tubes (Sarstedt) was used to perform adenosine diphosphate and thrombin receptor activating peptide tests. The area under the aggregation curve was used to determine overall platelet aggregation response.

### 2.6 Survival outcomes and prognostic associations

All patients were followed up for 360 days for a primary composite clinical outcome consisting of all-cause mortality (ACM), MI, and/or ischemic stroke. Secondary outcomes consisted of the single events ACM or MI. Acute MI and ischemic stroke were defined as described previously [24]. Follow-up was performed by telephonic interview and/or review of patients’ charts on readmission by investigators blinded to laboratory results. Forty-five (10.0%) patients were lost to follow-up.

### 2.7 Statistical analyses

All statistical analyses were performed using SPSS, version 27.0 (IBM), and GraphPad Prism software (GraphPad Software, Inc). Data are presented as median with 25th and 75th percentiles, mean ± SD, or count and percentage. Student’s *t*-tests and Mann–Whitney U-tests were applied as appropriate to analyze differences between the 2 groups. Analysis of variance tests were applied for comparison between >2 groups. Correlations of normally distributed data were assessed by Pearson rank correlation coefficient (r). Regression analyses were applied to test independent associations. Cox proportional hazard regression was applied to investigate the associations between survival outcomes and both platelet-associated and circulating CXCL14 levels using clinical factors as covariables. The time-dependent covariate method was used to check the proportional hazard assumption of the model. Survival functions were estimated by Kaplan-Meier curves. The log-rank test was applied to compare survival functions. All statistical tests were 2 tailed, and statistical significance level was defined as *P* < .05.

## 3 Results

### 3.1 Platelets as a potential source of CXCL14 in heart disease

**Figure 1:**
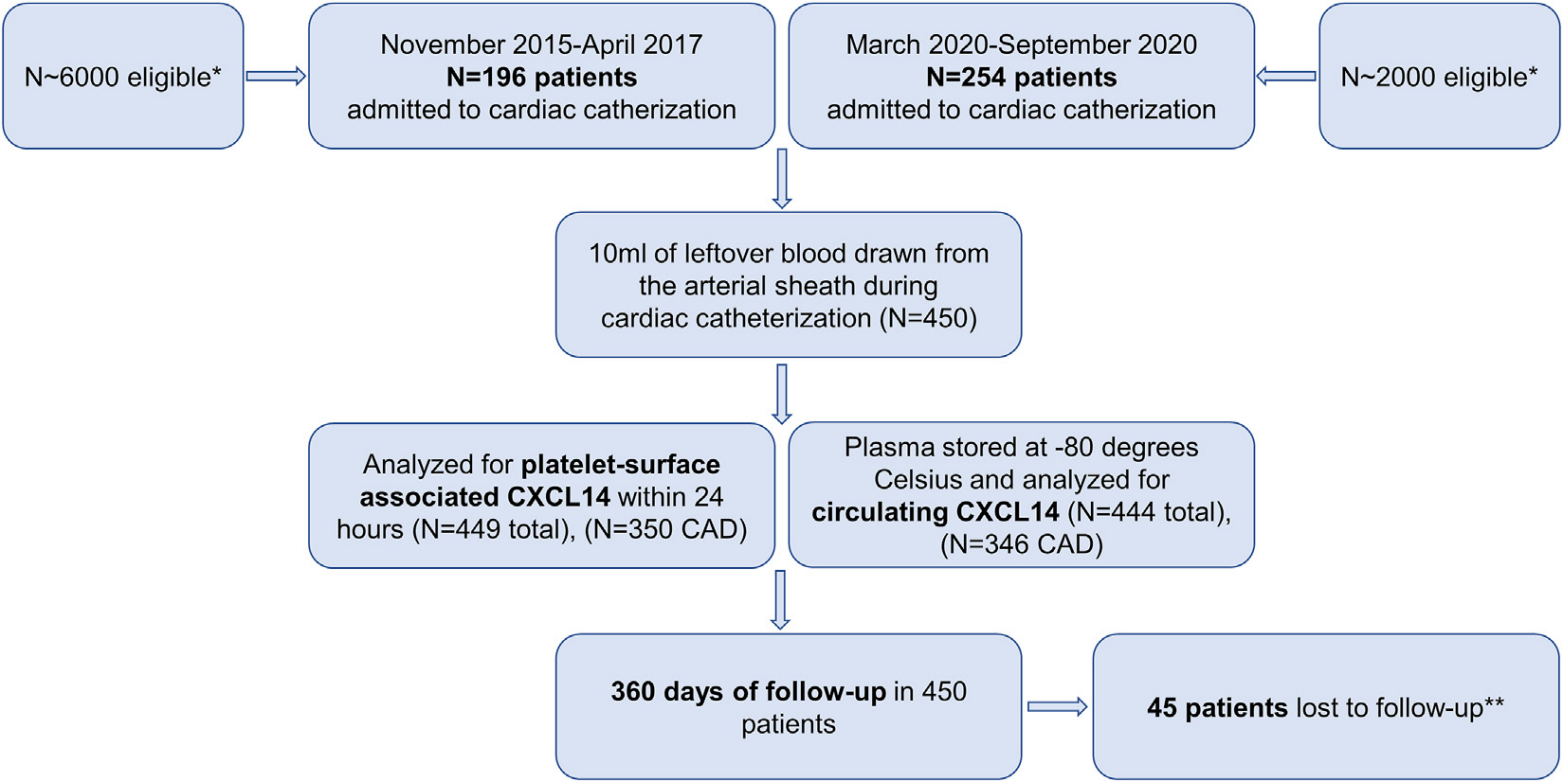
Study flow chart. CAD, coronary artery disease. ∗Due to staffing limitations and logistical challenges, we could not enroll more eligible patients. ∗∗Forty-five patients were lost to follow-up due to impossibility to establish contact to patients, treating physicians, or patients’ relatives.

**Figure 2:**
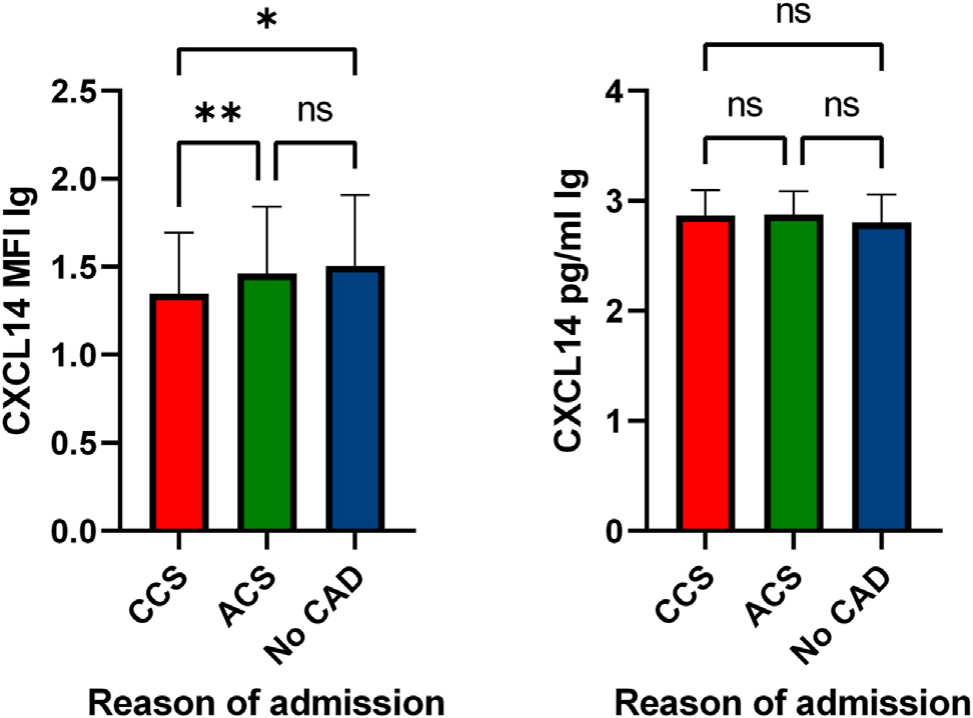
Baseline estimation of platelet surface–associated CXCL14 and circulating CXCL14 levels stratified according to chronic coronary syndrome, acute coronary syndrome, and noncoronary artery disease. ∗*P* < .05. ∗∗*P* < .01. ACS, acute coronary syndrome; CAD, coronary artery disease; CCS, chronic coronary syndrome; MFI, mean fluorescence intensity; ns, not significant.

**Figure 3:**
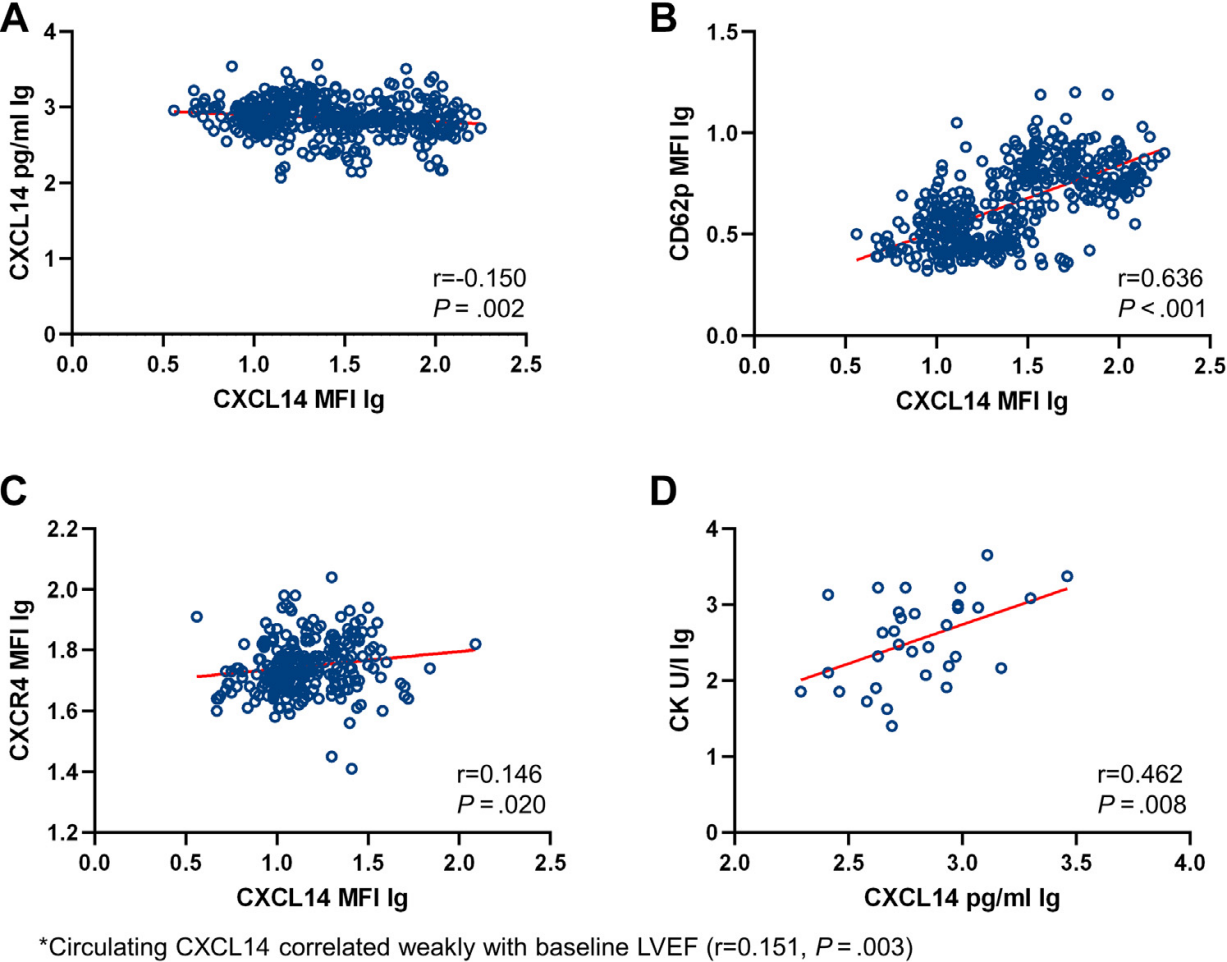
A, Correlation between baseline platelet surface–associated CXCL14 and circulating CXCL14 levels. B, Correlation between baseline CD62p platelet surface exposure and baseline CXCL14 platelet surface exposure. C, Correlation between baseline platelet surface–associated CXCL14 and baseline platelet surface–associated CXCR4 levels. D, Correlation between baseline creatine kinase and circulating CXCL14 levels. LVEF, left ventricular ejection fraction; MFI, mean fluorescence intensity.

The study flow chart is presented in Figure 1. We characterized the surface association of CXCL14 on circulating platelets and ascertained plasma levels of circulating CXCL14 in patients with symptomatic heart disease. Baseline characteristics of the complete clinical cohort stratified according to CAD vs non-CAD are presented in Table 1. Patients without CAD displayed the highest platelet surface–associated levels of CXCL14, followed by patients with ACS and those with CCS, showing the lowest platelet CXCL14 levels (mean mean fluorescence intensity logarithmized, 1.51 ± 0.40 vs 1.47 ± 0.38 vs 1.35 ± 0.35, respectively) (*P* for other vs ACS, 1; other vs CCS, .012; and ACS vs CCS, .008, respectively). Although we observed a difference in platelet-associated CXCL14, circulating CXCL14 plasma levels between these patient groups did not vary to a significant extent (mean CXCL14 lg, 2.80 ± 0.26 pg/mL vs 2.87 ± 0.21 pg/mL vs 2.86 ± 0.24 pg/mL; *P* = .089) (Figure 2). Nevertheless, we found that platelet surface–associated CXCL14 correlated weakly and inversely with circulating CXCL14 levels (n = 443, r = −0.150, *P* = .002). Furthermore, we observed a weak positive correlation between platelet-associated CXCL14 and platelet CXCR4 surface exposure (n = 252, r = 0.146, *P* = .020). Platelet surface–associated CXCL14 correlated significantly with the platelet activation marker and indicator of degranulation from α-granules CD62P (n = 447, r = 0.636, *P* < .001). Finally, in patients with STEMI, circulating CXCL14 correlated significantly with creatine kinase (CK), a marker of myocardial necrosis (n = 32, *r* = 0.462, *P* = .008) (Figure 3). MFIs of CXCR4 and CD62P as well as the concentrations of CK stratified according to CCS, ACS, and non-CAD are presented in Supplementary Table S1. Confounding, ie, association of covariates with the predictor variables (platelet surface–associated CXCL14 and circulating CXCL14) and outcome variables (ACM, MI, and combined outcome) was present only for age as a confounder of the relationship between circulating CXCL14 and ACM, diabetes for the relationship between platelet surface–associated CXCL14 and ACM and the combined outcome, and left ventricular ejection fraction (LVEF) for the relationship between platelet surface–associated CXCL14 and circulating CXCL14 and ACM. All of these confounders were adjusted for in the models between the respective predictors and outcome variables (Supplementary Tables S2 and S3).

**Table 1:** Baseline characteristics of the complete cohort.

Variables	CCS (n = 177)	ACS (n = 211) (STEMI n = 35; NSTEMI n = 70; unstable angina n = 106)	Other (n = 62)
Age, y (mean ± SD) (0 missing)	73 ± 10	70 ± 11	69 ± 12
Sex (male) (0 missing)	143 (80.7%)	156 (73.9%)	36 (58.1%)
LVEF (mean ± SD), % (58 missing)	52.8 ± 10.3	53.1 ± 10.2	51.5 ± 10.8
Ethnicity (0 missing)	176 White (99.4%)/1 Asian (0.6%)	210 White (99.5%)/1 Asian (0.5%)	62 White (100%)
CVRF (yes/no) (2 missing)			
Arterial hypertension	154 (87.0%)	178 (84.4%)	44 (71.0%)
Hyperlipidemia	113 (63.8%)	118 (55.9%)	26 (41.9%)
Diabetes	60 (33.9%)	52 (24.6%)	11 (17.7%)
Active smoking	65 (36.7%)	66 (31.3%)	5 (8.1%)
Medication at admission (yes/no) (25 missing)			
Acetylsalicylic acid	77 (43.5%)	88 (41.7%)	7 (11.3%)
Clopidogrel	21 (11.9%)	21 (10.0%)	1 (1.6%)
Prasugrel	11 (6.2%)	3 (1.4%)	0 (0.0%)
Ticagrelor	10 (5.6%)	8 (3.8%)	0 (0.0%)
Oral anticoagulation	25 (14.1%)	28 (13.3%)	16 (25.8%)
ACE inhibitors	37 (20.9%)	47 (22.3%)	8 (12.9%)
AT1 blockers	46 (26.0%)	46 (21.8%)	15 (24.2%)
Ca-channel blockers	24 (13.6%)	32 (15.2%)	11 (17.7%)
β-Blockers	78 (44.1%)	83 (39.3%)	26 (41.9%)
Thiazides	20 (11.3%)	21 (10.0%)	6 (9.7%)
Statins	86 (48.6%)	90 (42.7%)	17 (27.4%)
Sacubitril/Valsartan	5 (2.8%)	1 (0.5%)	1 (1.6%)
SGLT2 inhibitors	9 (5.1%)	1 (0.5%)	0 (0.0%)
Laboratory at admission (mean ± SD)			
Platelet count (× 1000/μL) (10 missing)	232 ± 64	233 ± 85	230 ± 71
White blood cell count (1/μL) (19 missing)	7684 ± 2343	8762 ± 3684	7298 ± 2112
Mean platelet volume (fL) (12 missing)	10.2 ± 1.0	10.2 ± 1.0	10.1 ± 0.9

ACE, angiotensin-converting enzyme; ACS, acute coronary syndrome; AT1, angiotensin 2 type 1; Ca, calcium; CCS, chronic coronary syndrome; CVRF, cardiovascular risk factor; LVEF, left ventricular ejection fraction; NSTEMI, non–ST-segment elevation myocardial infarction; SGLT2, sodium–glucose-linked transporter 2; STEMI, ST-segment elevation myocardial infarction.

### 3.2 Prognostic significance of platelets and circulating CXCL14

**Figure 4:**
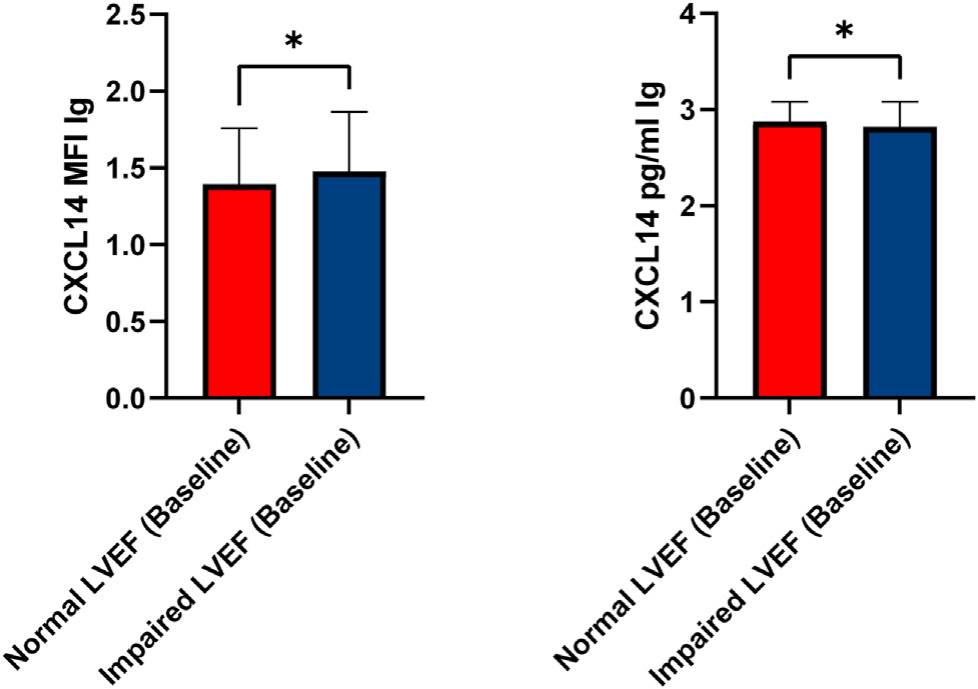
Baseline platelet surface–associated CXCL14 and circulating CXCL14 levels stratified according to normal vs impaired left ventricular function at study inclusion. LVEF, left ventricular ejection fraction; MFI, mean fluorescence intensity.

We assessed LVEF in 381 patients on admission, of whom 149 (normal, n = 88; impaired, n = 61) had a diagnosis of CCS, 179 (normal, n = 108; impaired, n = 71) had ACS, and 53 (normal, n = 27; impaired, n = 26) had no CAD. Both platelet surface–associated CXCL14 (mean MFI lg, 1.37 ± 0.37 vs 1.48 ± 0.39; *P* = .006) and circulating CXCL14 levels (mean CXCL14, 2.88 ± 0.20 pg/mL vs 2.82 ± 0.26 pg/mL; *P* = .029) differed significantly between patients with normal systolic LVEF and those with impaired systolic LVEF at admission (Figure 4).

**figure 5:**
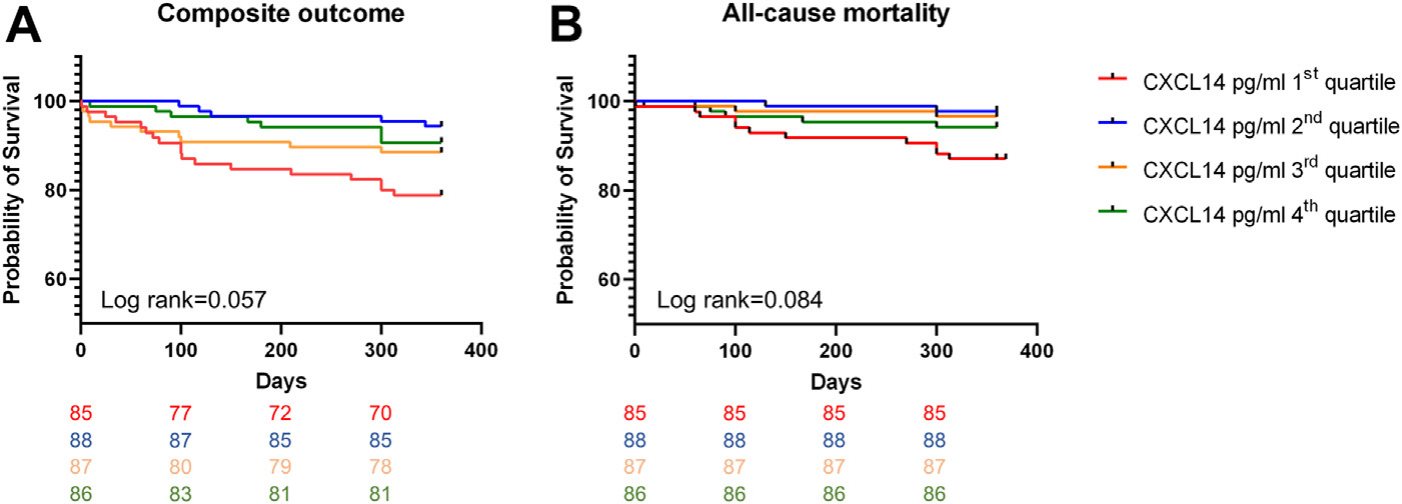
A, Kaplan-Meier curve showing worse event-free survival (composite outcome) in patients with low circulating CXCL14 levels at study inclusion. B, Kaplan-Meier curve showing worse event-free survival (all-cause mortality) in patients stratified according to circulating CXCL14 at study inclusion.

For the CAD cohort (n = 388), the number of events and incidence rates per 100 person-years are presented in Table 2. CXCL14 platelet surface–associated levels were not associated with outcome. On the other hand, there was a marked trend among patients with low circulating CXCL14 levels to have worse outcomes (composite outcome and ACM) when compared with those with higher circulating CXCL14 levels (Figure 5 and Table 3). As we did not see clear linear trends, we had to analyze the respective CXCL14 as categorical data. We, thus, had to primarily refer to the overall test with 3 degrees of freedom. In these tests, we did not find significant results. The results of multivariable Cox regression analyses are presented in Table 3. Data on the relative percentage of CXCL14-positive platelets are presented in Supplementary Information.

**Table 2:** Event rates and incidence rates per 100 person-years in patients with coronary artery disease.

Event^a^	CXCL14 MFI lg first quartile^b^	CXCL14 MFI lg second quartile^c^	CXCL14 MFI lg third quartile^d^	CXCL14 MFI lg fourth quartile^e^	Log-rank *P*
Composite outcome	10/88/11.4 (5.5/21.0)	9/89/10.1 (4.6/19.2)	10/86/11.6 (5.6/21.3)	12/87/13.8 (7.1/24.1)	.88
All-cause mortality	7/88/8.0 (3.2/16.5)	6/89/6.7 (2.5/14.6)	4/86/4.7 (1.3/12.0)	4/87/4.6 (1.3/11.8)	.74
Myocardial infarction	2/88/2.3 (0.3/8.3)	3/89/3.4 (0.7/9.9)	7/86/8.1 (3.3/16.7)	4/87/4.6 (1.3/11.8)	.29
Ischemic stroke	1/88/1.1 (0.0/6.1)	0/89/0.0 (0.0/4.1)	1/86/1.2 (0.0/6.7)	4/87/4.6 (1.3/11.8)	.11
Event	CXCL14 pg/mL lg first quartile^f^	CXCL14 pg/mL lg second quartile^g^	CXCL14 pg/mL lg third quartile^h^	CXCL14 pg/mL lg fourth quartile^i^	Log-rank *P*
Composite outcome	18/85/21.2 (12.6/33.5)	5/88/5.7 (1.9/13.3)	10/87/11.5 (5.5/21.2)	8/86/9.3 (4.0/18.3)	.057
All-cause mortality	11/85/12.9 (6.5/23.1)	2/88/2.3 (0.3/8.3)	3/87/3.4 (0.7/9.9)	5/86/5.8 (1.9/13.5)	.084
Myocardial infarction	7/85/8.1 (3.3/16.7)	2/88/3.5 (0.4/12.6)	5/87/5.8 (1.9/13.5)	2/86/2.3 (0.3/8.3)	.16
Ischemic stroke	2/85/2.4 (0.3/8.7)	1/88/1.1 (0.0/6.1)	2/87/2.3 (0.3/8.3)	1/86/1.2 (0.0/6.7)	.86

a Column 1 shows the different events. Columns 2 to 5 show the number of events/number at risk/incidence rate per 100 person-years with 95% CI in CXCL14 quartiles 1 to 4. The log-rank *P* values in column 6 show associations of CXCL14 grouped into quartiles with the respective outcomes. (In the cohort with coronary heart disease, due to technical issues, platelet CXCL14 was analyzed in 350 patients and circulating CXCL14 in 346 patients.).

b First quartile = x < 1.10.

c Second quartile ≥ 1.10 x < 1.40.

d Third quartile ≥ 1.40 x < 1.70.

e Fourth quartile x ≥ 1.70.

f First quartile = x < 2.75.

g Second quartile ≥ 2.75 x < 2.85.

h Third quartile ≥ 2.85 x < 3.00.

i Fourth quartile x ≥ 3.00.

**Table 3:** Results of multivariable Cox proportional hazard regression analyses with the composite outcome, all-cause mortality, and myocardial infarction as dependent variables, circulating CXCL14 and platelet surface–associated CXCL14 as prognostic markers, and clinical factors as covariates.^a^

Outcome	Circulating CXCL14 (fourth quartile vs first quartile)	Circulating CXCL14 (third quartile vs first quartile)	Circulating CXCL14 (second quartile vs first quartile)
Combined outcome	2.53 (1.09-5.89)	0.98 (0.37-2.63)	1.24 (0.49-3.14)
*P* = .054	*P* = .031	*P* = .98	*P* = .65
All-cause mortality	2.05 (0.70-6.01)	0.94 (0.27-3.24)	0.59 (0.14-2.46)
*P* = .19	*P* = .19	*P* = .94	*P* = .47
Myocardial infarction	3.92 (0.81-18.9)	0.95 (0.13-6.76)	2.59 (0.50-13.36)
*P* = .18	*P* = .089	*P* = .96	*P* = .26
Outcome	Platelet CXCL14 (first quartile vs fourth quartile)	Platelet CXCL14 (second quartile vs fourth quartile)	Platelet CXCL14 (third quartile vs fourth quartile)
Combined outcome	0.74 (0.32-1.73)	0.76 (0.33-1.58)	0.84 (0.37-1.90)
*P* = .89	*P* = .49	*P* = .52	*P* = .67
All-cause mortality	1.61 (0.47-5.49)	1.49 (0.43-5.12)	1.18 (0.32-4.38)
*P* = .87	*P* = .54	*P* = .53	*P* = .81
Myocardial infarction	0.53 (0.10-2.93)	0.90 (0.20-4.08)	1.62 (0.47-5.23)
*P* = .53	*P* = .47	*P* = .89	*P* = .44

a Adjustment factors: age, sex, acute coronary syndrome versus chronic coronary syndrome, arterial hypertension, hyperlipidemia, diabetes, smoker versus nonsmoker, ex-smoker vs nonsmoker, and left ventricular ejection fraction.

#### 3.2.1 Additional statistical testing/analyses.

Table 1 shows differences in baseline characteristics stratified according to CCS, ACS, and non-CAD. No other parameters were included for the statistical tests in Table 1. Table 2 shows event rates according to CXCL14 levels. No other parameters were included here. Cox regression analyses were applied to test independent associations of circulating CXCL14 with the composite outcome and ACM. No Cox regression analyses were generated for MI and ischemic stroke. No additional testing was performed for Supplementary Tables S1 and S2. For Figure 2, no additional testing was performed. Additional testing was performed in addition to the results presented in Figures 3 and 4. We additionally verified correlations of platelet-associated CXCL14-MFI with PAC-1-MFI, CXCL14 and troponin I, CXCL14, and LVEF. Circulating CXCL14 correlated weakly with baseline LVEF (*r* = 0.151, *P* = .003). We could not find significant correlations between the other parameters (data not shown). Kaplan-Meier curves were generated for high vs low platelet-associated CXCL14 as well as for low vs high circulating CXCL14 to evaluate their prognostic association with composite outcome, ACM, and MI. As mentioned previously, platelet-associated CXCL14 was neither associated with the composite outcome, ACM, nor MI. Finally, we investigated the platelet aggregation profile in Multiplate impedance aggregometry (ADP and TRAP) and its possible associations with platelet CXCL14 stratified according to different P_2_Y_12_ inhibitor treatments (Supplementary Figure).

## 4 Discussion

The current investigation revealed that (1) the CXCL14 platelet surface association was higher in patients with ACS and patients without CAD when compared with those with CCS; (2) both platelet-associated and circulating CXCL14 levels differed in patients with normal vs impaired baseline systolic LVEF; and (3) only low circulating CXCL14 (first quartile) may be associated with worse outcomes in patients with CAD, whereas there were no relevant correlations for platelet-associated CXCL14 [5]. However, platelet surface–associated CXCL14 correlated with the extent of platelet activation (CD62P) in patients with CAD, while plasma CXCL14 correlated with myocardial necrosis in patients with STEMI [6,7]. Finally, the observed associations remained statistically significant after adjustment for confounders.

Previous studies and experiments have suggested that CXCL14 may play a role in the pathophysiology of CAD [4,5]. Activated platelet–derived CXCL14 shows prominent thromboinflammatory influence in triggering monocyte migration [4,5,15], while it also exerts an angiostatic effect on endothelial cells and counteracts the angiogenic response of vascular endothelial growth factor and CXCL12 [4], which may impair vascular regeneration or re-endothelialization after MI. These proinflammatory and angiostatic properties of CXCL14 taken together may foster progression of atherosclerosis, eventually leading to CAD, and also hinder functional recovery of the affected myocardium following ischemia-reperfusion injury by retarding angiogenesis. Therefore, we sought to investigate platelet-associated and circulating CXCL14 levels in patients with heart disease in this translational study. We found that platelet surface–associated CXCL14 but not circulating CXCL14 levels were differentially regulated in patients with CCS when compared with those in patients with ACS and in patients with CCS when compared with those in patients without CAD. It is noteworthy that cellular sources other than platelets may contribute to plasma CXCL14, particularly in patients with heart disease predisposed to a chronic inflammatory state. Nevertheless, activated platelets are a major source of chemokines that are released upon stimulation [[25], [26], [27]], explaining the correlation between platelet surface–associated CXCL14 and their activation status, particularly CD62P surface expression, denoting degranulation from α-granules. Furthermore, this justifies lower platelet-associated CXCL14 levels in patients with CCS who are more frequently treated with antiplatelet drugs, regulating their platelet activation status as compared with those in patients with ACS and without CAD. Patients with ACS are expected to be treated more often with ticagrelor or prasugrel compared with patients with CCS. However, because ACS is often the first clinical manifestation of CAD, many patients are not pretreated with P_2_Y_12_ inhibitors. On the other hand, a considerable number of patients who initially had ACS and those with CCS, eg, 6 months after the index event, may still receive P_2_Y_12_ inhibitors following the initial prescription. Thus, blocking platelet activation with acetylsalicylic acid and/or P_2_Y_12_ inhibitors may presumably diminish platelet chemokine release. Therefore, we reinvestigated the aggregation profile of 446 patients after ADP and TRAP stimulation. Considering that ADP does not activate platelets to the extent that TRAP does, besides TRAP-induced aggregation cannot be completely abolished by P_2_Y_12_ inhibitors, unlike ADP, we created a ratio between ADP- and TRAP-induced aggregation. A higher ratio denotes that platelets are more prone to ADP activation. In the overall cohort, we observed higher platelet-associated CXCL14 levels in patients exhibiting a higher ADP/TRAP ratio. However, we could not find significant differences in platelet-associated CXCL14 levels when patients with CCS were stratified according to treatment with clopidogrel, ticagrelor, or prasugrel. Interestingly, we found a much lower ADP/TRAP ratio in patients taking the more potent P_2_Y_12_ inhibitors ticagrelor or prasugrel when compared with those taking clopidogrel, suggesting better inhibition of platelet activation. In patients with potent P_2_Y_12_ inhibitors, we could show a strong correlation between ADP/TRAP ratio and platelet-associated CXCL14, indirectly suggesting that strong inhibition of platelet activation with potent P_2_Y_12_ inhibitors results in lower platelet-associated CXCL14 levels (Supplementary Figure). However, besides platelets, circulating leukocytes and inflamed endothelium may contribute significantly to circulatory CXCL14 levels. [3,15,28] Platelet-associated CXCL14 and circulating CXCL14 correlated inversely, which may indicate that platelets bind circulating CXCL14 onto their surface. Hyperactive platelets may act as a prime source of circulatory CXCL14, particularly in ACS, as they engage in mounting acute myocardial inflammation and subsequent regenerative or fibrotic processes following MI [29].

Unexpectedly, low circulating CXCL14 levels at baseline were associated with worse event-free survival. However, similar to LVEF, prognosis may be favorable if circulating CXCL14 levels decline over time. Intriguingly, circulating CXCL14 levels at baseline were lower in patients with ACS and impaired LVEF when compared with those with normal LVEF, which may have resulted from a counterregulatory mechanism to downregulate angiostatic circulating CXCL14 levels to foster angiogenesis and myocardial regeneration. Because ACS with impaired LVEF usually corresponds to a worse prognosis, this may explain the observed effects of CXCL14 on prognosis. Speculatively, circulating CXCL14 below a certain threshold level may adversely affect outcomes. Finally, in patients with STEMI, circulating CXCL14 levels correlated with the extent of myocardial necrosis (CK), suggesting that hypoxia may increase the expression of CXCL14 [30] and influence the release of this chemokine during the acute phase response. Either platelet surface–associated or circulating CXCL14 or both may exert a potential influence on platelet responsiveness by acting through its receptor CXCR4 [5]. Despite previous controversies regarding the possibility of CXCR4 being the cognate receptor for CXCL14, we have demonstrated that CXCL14 can specifically bind to platelet CXCR4 [5] and currently observed a correlation between platelet surface–associated CXCL14 and CXCR4 in a clinical cohort. Elevated CXCR4 expression on platelets in patients with CAD may, therefore, influence platelet CXCL14 binding, which is also more likely to be altered by epidemiologic and risk factors than circulating CXCL14. For example, sex and smoking habits showed independent associations with platelet CXCL14 in most multivariable analyses, while circulating CXCL14 seemed to be less influenced by these factors.

To conclude, we provide evidence on the differential regulation of platelet-associated and circulating CXCL14 levels and their association with myocardial function in patients with heart disease. Finally, we demonstrate associations of CXCL14 with the prognosis of patients with CAD. These findings provide interesting insights into the barely investigated effects of CXCL14 on the cardiovascular system and encourage further research in this direction.

### 4.1 Study limitations

Being a translational study, adequate explanations for the clinical findings remain to be verified through experimental investigations in CXCL14 deficient cellular systems (ie, iPS-derived platelets) or murine models (eg, megakaryocyte-platelet lineage-specific CXCL14-deficient mice). Platelets are not the exclusive source of circulating CXCL14 and could be derived from circulating leukocytes; this was not explored in the current cohort and must be attended to in future studies. Another limitation of the current study is that angiogenesis was not investigated. Although we did observe a correlation between platelet surface–associated CXCL14 and platelet surface expression of its receptor CXCR4, we did not verify this association with biochemical evidence from the current clinical cohort as we have previously demonstrated in our experimental studies through confocal microscopy, coimmunoprecipitation, and human iPS culture–derived platelets [5]. Such experimental analyses would have required copious amounts of blood, which could not be attempted due to ethical limits. Furthermore, the sample size of our cohort was moderate, which impaired the power to investigate secondary outcomes. We could not provide sequential biomarker measurements for the complete study collective, which would have been highly desirable to further delineate the chronic effects of platelet-associated and circulating CXCL14 on prognosis in CAD. In addition, the current study has several possible sources of bias, including a selection bias, since only a minority of eligible patients was enrolled in the study. Ten percent of patients were lost to follow-up. To our knowledge, we considered all possible confounders and adjusted for them. However, we cannot exclude residual confounding by unknown variables not included in our analysis. Furthermore, we performed 6 analyses with 2 predictors (platelet surface–associated and circulating CXCL14) and 3 outcomes (combined outcome, ACM, and MI). The Bonferroni correction was not feasible due to the limited power of the study. Thus, we cannot exclude the findings by chance.

## Supplementary Material

Supplemental Material **Supplementary Table S1**. MFIs of CXCR4 and CD62p as well as U/l for CK stratified according to CCS, ACS, and no CAD. **Supplementary Table S2**. Multivariable analyses with platelet and circulating CXCL14 as dependent variables and cardiovascular risk factors as covariates. Multivariable models were applied to all univariable data presented in Figures 2-4. The blue rows indicate dependent variables, whereas white rows indicate independent variables. The multivariable models are in the same order as the univariable models in the figures. Respective figures to which the multivariable models refer are presented in the blue rows containing the dependent variables. **Supplementary Table S3.** Potential Confounder p-values (association with predictors and outcomes) **Supplementary figure 1:A.** Box-plots showing platelet-associated CXCL14 stratified according to ADP/TRAP ratio. AUC=Area under the curve. **B.** Box-plots showing ADP/TRAP ratio stratified according to clopidogrel vs. ticagrelor/prasugrel treatment. **C.** Scatter-plot showing a correlation between platelet-associated CXCL14 and ADP/TRAP ratio.
