# Supplementary material for: Platelet versus plasma CXCL14, coronary artery disease, and clinical outcomes
Source: Res Pract Thromb Haemost. 2023 Apr 25;7(4):100165. doi: 10.1016/j.rpth.2023.100165 (PMC10225916; doi:10.1016/j.rpth.2023.100165)

**Supplementary information:**

Results on flow-cytometry given as % of CXCL14 positive platelets:

Patients without CAD displayed the highest platelet surface association of CXCL14, followed by ACS patients and those with CCS showing the lowest platelet CXCL14 levels [Median % of CXCL14+ platelets 63.2, 25th/75th percentile 11.5/78.4 vs 59.0, 25th/75th percentile 9.9/74.9 vs 34.2, 25th/75th percentile 7.3/68.9] (p for other vs ACS 0.225, other vs CCS 0.005 and ACS vs CCS 0.021, respectively). % of CXCL14 positive platelets correlated weakly and inversely with circulating CXCL14 levels (rho=-0.143, p=0.003). % of CXCL14 positive platelets correlated weakly with platelet CXCR4 surface exposure (rho=0.176, p=0.005). Furthermore, % of CXCL14 positive platelets correlated significantly with CD62P (rho=0.582, p<0.001). Platelet surface-associated CXCL14 [Median MFI 42.5, 25th/75th percentile 6.4/71.2 vs 58.0, 25th/75th percentile 11.1/76.2, p=0.011] differed significantly in patients with normal vs impaired LVEF at admission. Finally, patients with high % of CXCL14+ platelets (4th quartile) did not show a worse cumulative event-free survival for the CE when compared to patients with lower levels (1st-3rd quartile) (Log rank p=0.260).

**Supplementary table 1:**

|  | **CCS** | **ACS** | **Other** |
| --- | --- | --- | --- |
| **CXCR4 MFI (n=252)** | 36.0 (29.2/47.0) | 34.2 (27.2/41.5) | 33.1 (26.7/40.2) |
| **CD62p MFI (n=447)** | 3.7 (2.8/5.9) | 5.2 (3.2/7.1) | 5.0 (3.4/6.4) |
| **CK U/l (n=360)** | 98.5 (67.0/151.0) | 112.0 (75.0/209.0) | 102 (68/175) |

**Supplementary table 2:**

| **Dependent variable  CXCL14 MFI lg (Figure 2)** | **Regression coefficient  (95% CI)** |
| --- | --- |
| Age | 0.001 (-0.003-0.004) |
| Gender | 0.13 (0.53-0.21) |
| Arterial hypertension | -0.11 (-0.20- -0.01) |
| Hyperlipidemia | 0.03 (-0.04-0.10) |
| Diabetes | -0.04 (-0.11-0.04) |
| Smoker vs Non-smoker | -0.12 (-0.20- -0.05) |
| Ex-smoker vs Non-smoker | 0.32 (0.18-0.46) |
| ACS vs CCS | 0.11 (0.04-0.18) |
| No CAD vs CCS | 0.14 (0.04-0.25) |
| **Dependent variable  Circulating CXCL14 lg (Figure 2)** | **Regression coefficient  (95% CI)** |
| Age | -0.0020 (-0.0041-0.0001) |
| Gender | -0.01 (0.07-0.04) |
| Arterial hypertension | -0.03 (-0.10- 0.03) |
| Hyperlipidemia | 0.01 (-0.04-0.06) |
| Diabetes | -0.01 (-0.06-0.04) |
| Smoker vs Non-smoker | -0.01 (-0.04-0.06) |
| Ex-smoker vs Non-smoker | -0.08 (-0.17-0.01) |
| ACS vs CCS | 0.01 (-0.04-0.06) |
| No CAD vs CCS | -0.07086 (-0.14175-0.00003) |
| **Dependent variable  CXCL14 MFI lg (Figure 3 A)** | **Regression coefficient  (95% CI)** |
| Age | 0.00003 (-0.00317-0.00323) |
| Gender | 0.12 (0.05-0.20) |
| Arterial hypertension | -0.11 (-0.22- -0.02) |
| Hyperlipidemia | 0.03 (-0.04-0.10) |
| Diabetes | -0.03 (-0.11-0.04) |
| Smoker vs Non-smoker | -0.12 (-0.20- -0.05) |
| Ex-smoker vs Non-smoker | 0.30 (0.17-0.44) |
| ACS vs CCS | 0.12 (0.04-0.19) |
| No CAD vs CCS | 0.13 (0.02-0.24) |
| Circulating CXCL14 lg | -0.21 (-0.36- -0.07) |
| **Dependent variable  CXCL14 MFI lg (Figure 3 B)** | **Regression coefficient  (95% CI)** |
| Age | 0.001 (-0.001-0.004) |
| Gender | 0.11 (0.05-0.18) |
| Arterial hypertension | -0.07 (-0.15-0.01) |
| Hyperlipidemia | 0.04 (-0.02-0.09) |
| Diabetes | -0.03 (-0.09-0.03) |
| Smoker vs Non-smoker | -0.06 (-0.12-0.01) |
| Ex-smoker vs Non-smoker | 0.15 (0.04-0.27) |
| ACS vs CCS | 0.02 (-0.04-0.08) |
| No CAD vs CCS | 0.09 (0.04-0.18) |
| CD62p MFI lg | 1.14 (0.99-1.28) |
| **Dependent variable  CXCL14 MFI lg (Figure 3 C)** | **Regression coefficient  (95% CI)** |
| Age | 0.001 (-0.002-0.003) |
| Gender | 0.04 (-0.03-0.11) |
| Arterial hypertension | -0.06 (-0.15-0.03) |
| Hyperlipidemia | -0.01 (-0.07-0.06) |
| Diabetes | -0.04 (-0.10-0.03) |
| Smoker vs Non-smoker | 0.03 (-0.04-0.09) |
| ACS vs CCS | -0.03 (-0.09-0.04) |
| No CAD vs CCS | -0.00 (-0.10-0.09) |
| CXCR4 MFI lg | 0.35 (0.02-0.68) |
| **Dependent variable  Circulating CXCL14 lg (Figure 3 D)** | **Regression coefficient  (95% CI)** |
| CK U/l lg | 0.21 (0.06-0.36)* |
| **Dependent variable  CXCL14 MFI lg (Figure 4)** | **Regression coefficient  (95% CI)** |
| Age | -0.001 (-0.005-0.002) |
| Gender | 0.10 (0.01-0.18) |
| Arterial hypertension | -0.10 (-0.20- 0.01) |
| Hyperlipidemia | 0.02 (-0.06-0.09) |
| Diabetes | -0.02 (-0.11-0.06) |
| Smoker vs Non-smoker | -0.15 (-0.23- -0.06) |
| Ex-smoker vs Non-smoker | 0.31 (0.16-0.45) |
| ACS vs CCS | 0.10 (0.02-0.18) |
| No CAD vs CCS | 0.17 (0.06-0.29) |
| LVEF normal vs impaired | 0.076 (0.003-0.150) |
| **Dependent variable  Circulating CXCL14 lg (Figure 4)** | **Regression coefficient  (95% CI)** |
| Age | -0.002 (-0.004-0.001) |
| Gender | 0.02 (-0.03-0.08) |
| Arterial hypertension | -0.05 (-0.11-0.02) |
| Hyperlipidemia | 0.01 (-0.04-0.05) |
| Diabetes | -0.02 (-0.07-0.04) |
| Smoker vs Non-smoker | 0.01 (-0.05-0.06) |
| Ex-smoker vs Non-smoker | -0.11 (-0.21-0.02) |
| ACS vs CCS | 0.01 (-0.04-0.06) |
| No CAD vs CCS | -0.09 (-0.17- -0.01) |
| LVEF normal vs impaired | -0.051 (-0.099- -0.004) |

*Forward variable selection due to low number of cases

**Supplementary table 3:**

| Potential. Confounder | Predictors | | Outcomes | | |
| --- | --- | --- | --- | --- | --- |
|  | Platelet CXCL14 | Circulating CXCL14 | Combined | All-cause Mortality | Myocardial Infarction |
| Age | 0.52 | 0.047 | 0.351 | 0.019 | 0.596 |
| Gender | 0.004 | 0.901 | 0.903 | 0.891 | 0.567 |
| Arterial hypertension | 0.004 | 0.320 | 0.262 | 0.374 | 0.322 |
| Hyperlipidemia | 0.671 | 0.687 | 0.023 | 0.065 | 0.091 |
| Diabetes | 0.066 | 0.629 | 0.011 | 0.027 | 0.136 |
| Smoking | <0.001 | 0.123 | 0.249 | 0.906 | 0.586 |
| Reason of admission | 0.002 | 0.089 | 0.105 | 0.671 | 0.113 |
| LVEF | 0.006 | 0.022 | 0.219 | 0.070 | 0.220 |

Red: p≤0.05, green: p≤0.10

**Supplementary figure 1:**

A


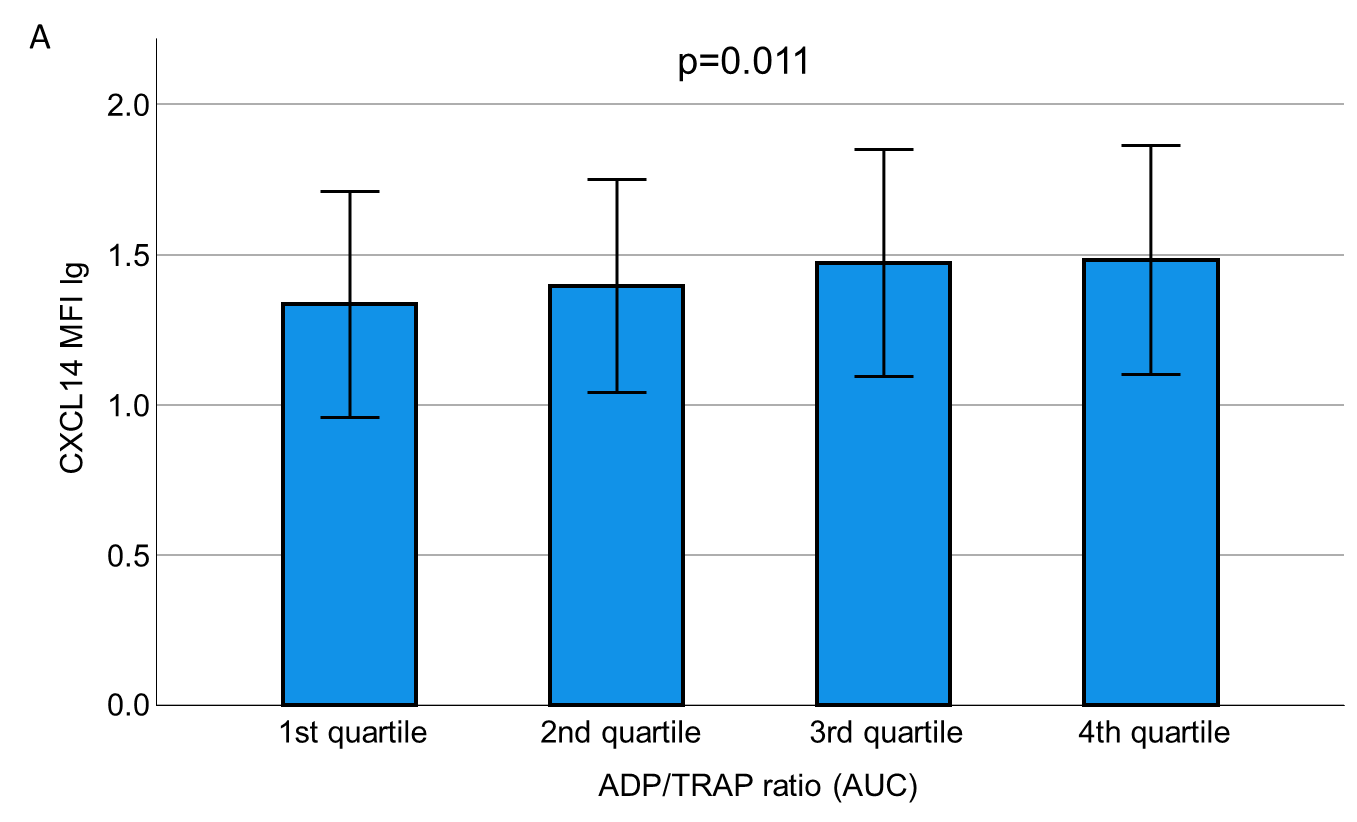


C

B


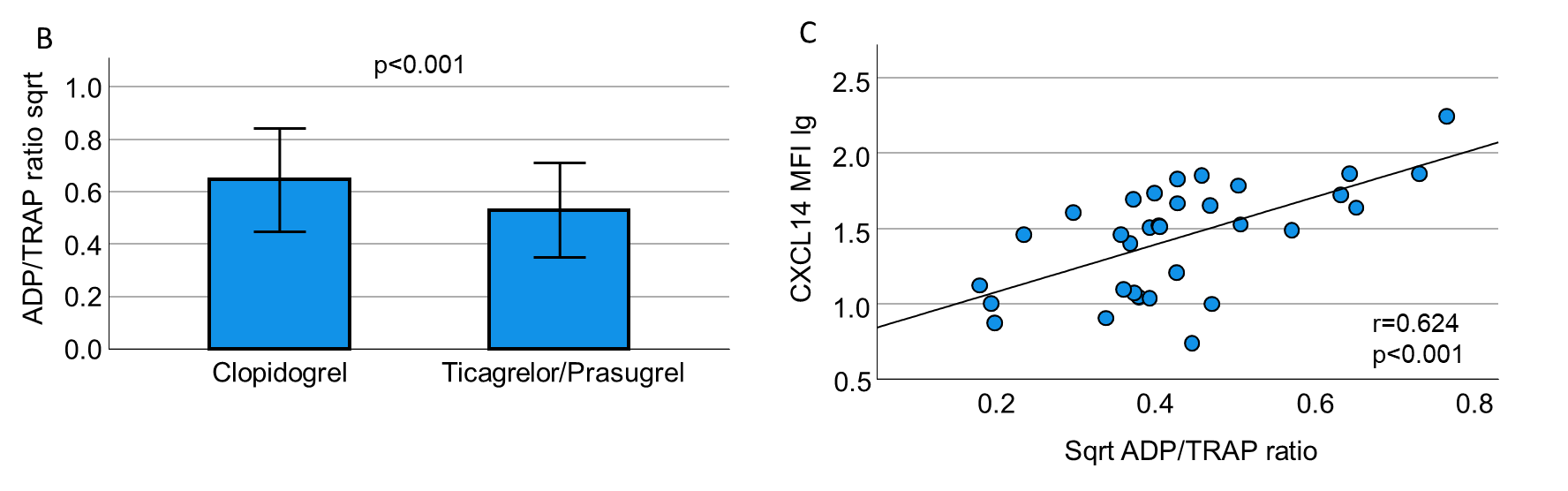

Supplement: Supplemental Material — Supplementary Table S1. MFIs of CXCR4 and CD62p as well as U/l for CK stratified according to CCS, ACS, and no CAD. Supplementary Table S2. Multivariable analyses with platelet and circulating CXCL14 as dependent variables and cardiovascular risk factors as covariates. Multivariable models were applied to all univariable data presented in Figures 2-4. The blue rows indicate dependent variables, whereas white rows indicate independent variables. The multivariable models are in the same order as the univariable models in the figures. Respective figures to which the multivariable models refer are presented in the blue rows containing the dependent variables. Supplementary Table S3. Potential Confounder p-values (association with predictors and outcomes) Supplementary figure 1:A. Box-plots showing platelet-associated CXCL14 stratified according to ADP/TRAP ratio. AUC=Area under the curve. B. Box-plots showing ADP/TRAP ratio stratified according to clopidogrel vs. ticagrelor/prasugrel treatment. C. Scatter-plot showing a correlation between platelet-associated CXCL14 and ADP/TRAP ratio. [file mmc1.docx]
